# Impaired HDL antioxidant and anti-inflammatory functions are linked to increased mortality in acute heart failure patients

**DOI:** 10.1016/j.redox.2024.103341

**Published:** 2024-09-05

**Authors:** Anja Pammer, Iva Klobučar, Julia T. Stadler, Sabine Meissl, Hansjörg Habisch, Tobias Madl, Saša Frank, Vesna Degoricija, Gunther Marsche

**Affiliations:** a Division of Pharmacology, Otto Loewi Research Center, Medical University of Graz, Graz, Austria; b Department of Cardiology, Sisters of Charity University Hospital Centre, Zagreb, Croatia; c Medicinal Chemistry, Otto Loewi Research Center, Medical University of Graz, Graz, Austria; d BioTechMed-Graz, Graz, Austria; e Division of Molecular Biology and Biochemistry, Gottfried Schatz Research Center, Medical University of Graz, Graz, Austria; f Department of Medicine, Sisters of Charity University Hospital Centre, Zagreb, Croatia; g School of Medicine, University of Zagreb, Zagreb, Croatia

**Keywords:** Acute heart failure, HDL, Antioxidant enzymes, PON1, LCAT, Cholesterol efflux capacity, Mortality

## Abstract

**Aims:**

Acute heart failure (AHF) is typified by inflammatory and oxidative stress responses, which are associated with unfavorable patient outcomes. Given the anti-inflammatory and antioxidant properties of high-density lipoprotein (HDL), this study sought to examine the relationship between impaired HDL function and mortality in AHF patients. The complex interplay between various HDL-related biomarkers and clinical outcomes remains poorly understood.

**Methods:**

HDL subclass distribution was quantified by nuclear magnetic resonance spectroscopy. Lecithin–cholesterol acyltransferase (LCAT) activity, cholesterol ester transfer protein (CETP) activity, and paraoxonase (PON-1) activity were assessed using fluorometric assays. HDL-cholesterol efflux capacity (CEC) was assessed in a validated assay using [3H]-cholesterol-labeled J774 macrophages.

**Results:**

Among the study participants, 74 (23.5 %) out of 315 died within three months after hospitalization due to AHF. These patients exhibited lower activities of the anti-oxidant enzymes PON1 and LCAT, impaired CEC, and lower concentration of small HDL subclasses, which remained significant after accounting for potential confounding factors. Smaller HDL particles, particularly HDL3 and HDL4, exhibited a strong association with CEC, PON1 activity, and LCAT activity.

**Conclusions:**

In patients with AHF, impaired HDL CEC, HDL antioxidant and anti-inflammatory function, and impaired HDL metabolism are associated with increased mortality. Assessment of HDL function and subclass distribution could provide valuable clinical information and help identify patients at high risk.

## Highlights

• HDL function, structure and metabolism in hospitalized AHF patients significantly differ between survivors and non-survivors.
• HDL function markers PON1, LCAT activity, CEC, and small HDL particles predict 3-month mortality as effectively as NT-proBNP
• NMR analysis of HDL subclasses reveals insights into HDL function, with potential for clinical use and targeted therapies.

## Graphical abstract

Impaired HDL function and metabolism are associated with an increased risk of mortality in patients with AHF. Evaluating HDL function, subclass distribution, and metabolism may offer valuable clinical insights for predicting prognosis in AHF patients. A total of 315 patients hospitalized due to AHF were evaluated at baseline. Serum HDL subclass levels were measured by nuclear magnetic resonance (NMR) spectroscopy. HDL cholesterol efflux capacity (CEC), paraoxonase 1 (PON1) activity, lecithin-cholesterol acyltransferase (LCAT) activity, and cholesterol ester transfer protein (CETP) activity were assessed using cell-based assays and commercially available kits. After accounting for potential confounding factors, lower levels of HDL-CEC, PON1 activity, LCAT activity, and smaller HDL subclasses remained significantly associated with an increased risk of death within three months.

**Figure:**
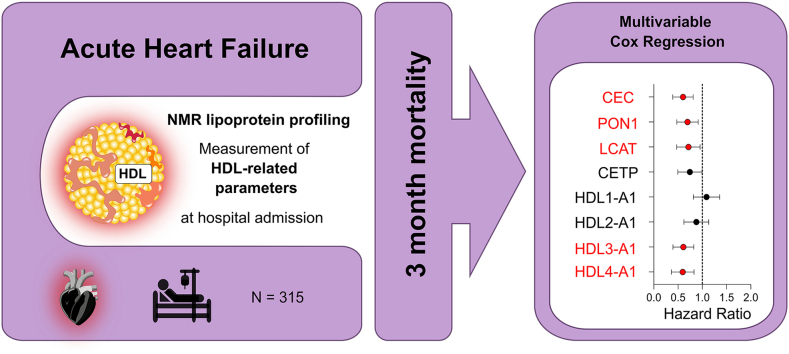

## 1 Introduction

Previous research has demonstrated that low levels of HDL particles are independent risk factors for both mortality and hospital readmissions due to recurrent symptoms of AHF [[1], [2], [3]]. Studies in animal models have demonstrated HDL-mediated favorable cardiac remodeling in pre-heart failure settings such as diabetic cardiomyopathy [4], post-myocardial infarction [5], and chronic pressure overload [6]. A single injection of reconstituted HDL given to mice shortly after myocardial infarction has shown therapeutic potential by increasing cardiac glucose uptake, protecting heart cells from death, and restoring cardiac function, effectively halting the progression to heart failure [7].

Oxidative stress is a fundamental pathophysiological mechanism closely associated with the aging process and is involved in heart failure's development and progression [8,9]. Various constituents of HDL, in particular its major protein constituent apolipoprotein A-I (apoA-I), the antioxidant enzyme paraoxonase 1 (PON1) and lecithin–cholesterol acyltransferase (LCAT) play a crucial role in the anti-oxidative and cardioprotective effects triggered by HDL [[10], [11], [12], [13]]. PON1 is an essential antioxidant component in HDL that is important in hydrolyzing oxidized lipids within lipoproteins [[14], [15], [16], [17]]. LCAT plays a vital role in the maturation of HDL particles and has a unique ability to break down oxidized short-chain phospholipids [18]. LCAT-targeted therapies have emerged as a promising approach for treating LCAT deficiency and potentially other cardiovascular diseases [19]. Small, dense HDL particles show the most potent antioxidative, anti-inflammatory, and antiapoptotic activities [20]. The ability of HDL to remove cholesterol and oxysterols from cells, better known as cholesterol efflux capacity (CEC), is a potent anti-atherogenic function of HDL [21]. By facilitating the removal of excess cholesterol from cardiac cells, HDL may help prevent lipid accumulation and cellular damage. By promoting the efflux of cholesterol and 7-oxysterols, HDL particles maintain endothelial function and preserve active eNOS dimer levels [22].

Despite previous research, the relationship between different HDL-related biomarkers and clinical outcomes remains incompletely understood. Furthermore, a direct comparison of their association with incident events is lacking. In this study, we investigated the relationship between various HDL parameters and the prognosis of patients who presented to the emergency department with severe signs and symptoms of AHF that required hospital treatment. We aimed to identify HDL markers indicative of quality and quantity associated with 3-month mortality following the index AHF hospitalization.

## 2 Methods

### 2.1 Study cohort

The AHF study is a prospective, observational, single-center study conducted over 36 months. It recruited consecutive adult patients who presented to the emergency department of the university hospital center with severe signs and symptoms of acute heart failure (AHF) requiring hospitalization. Patients with mild clinical signs and symptoms of heart failure who were appointed to ambulatory treatment, as well as hospitalized AHF patients with severe non-cardiovascular comorbidities and those who refused to participate were excluded (Supplemental Fig. 1).

At the time of presentation to the emergency department, a comprehensive patient history was documented for all participants. Physical and echocardiography examinations were conducted, and venous blood samples were collected for analysis before treatment. The follow-up period relevant to this substudy was three months post-index AHF hospitalization, with the primary endpoint being the participants' survival status at that time.

All patients who participated in the study provided written informed consent following the guidelines set forth by Good Clinical Practice. Furthermore, the study adhered to the principles outlined in the Declaration of Helsinki. Ethical approval was obtained from the local ethics committees of the Sisters of Charity University Hospital Centre in Zagreb, Croatia (EP 2258/18-10) and the Medical University of Graz in Austria (EC 33–258 ex 20/21).

### 2.2 ApoB-Depletion of serum

To analyze the composition and function of HDL, we utilized serum HDL (apoB-depleted serum) [23]. To prepare apoB-depleted serum, 40 μL polyethylene glycol (20 % in 200 mmol/L glycine buffer) (Sigma-Aldrich, Darmstadt, Germany) was gently mixed with 100 μL serum. The mixture was incubated at room temperature for 20 min, followed by centrifugation at 10,000×*g* for 30 min at 4 °C. The supernatant was then collected and samples were stored at −70 °C until required.

### 2.3 NMR spectroscopy measurements

Serum levels of HDL subclasses were measured on a Bruker 600 MHz Avance Neo NMR spectrometer using the Bruker IVDr lipoprotein subclass analysis protocol [24]. NMR spectra were recorded at a constant temperature of 310 K using various pulse sequences for proton spectra acquisition and water suppression. Data analysis for lipoprotein quantification was performed using the Bruker IVDr Lipoprotein Subclass Analysis (B.I.LISA™) method.

### 2.4 Cholesterol efflux capacity

The cholesterol efflux capacity was assessed as previously published [25,26] with a cell-based method. J774.2 cells (Sigma Aldrich, Darmstadt, Germany) were seeded at a density of 300,000 cells per well in 48-well plates and incubated for 24 h. The cells were labeled with 0.5 μCi/mL radiolabelled [3H]-cholesterol (Hartmann Analytic, Braunschweig, Germany) in DMEM media containing 2 % FBS, 1 % penicillin/streptomycin, and 8(4-chlorophenylthio)-cyclic adenosine monophosphate (0.3 mM) (Sigma-Aldrich, Darmstadt, Germany) overnight. After rinsing, cells were equilibrated for 2 h with serum-free DMEM containing 2 % bovine serum albumin (Sigma-Aldrich, Darmstadt, Germany), rinsed again, and incubated with 2.8 % apoB-depleted serum for 3 h. CEC was quantified by calculating the ratio of radioactivity in the media to the total radioactivity of the media and lysed cells.

### 2.5 LCAT activity

According to the manufacturer's protocol, serum LCAT activity was assessed using a commercial kit (MAK107, Merck, Darmstadt, Germany). Samples were incubated with the LCAT substrate for 4 h at 37 °C. The fluorescent substrate emits at 470 nm, and upon LCAT-mediated hydrolysis, a monomer with fluorescence at 390 nm is released. LCAT activity was measured by monitoring the change in the ratio of emission intensities at 470 nm and 390 nm over time.

### 2.6 Arylesterase activity of paraoxonase 1

The arylesterase activity of PON1 was evaluated utilizing a photometric assay with phenylacetate substrate, as outlined in the specified reference [27]. ApoB-depleted serum was added to a 200 μL buffer solution containing 100 mM Tris, 2 mM CaCl₂ (pH 8.0), and 1 mM phenylacetate. Phenylacetate hydrolysis was monitored at 270 nm. Enzymatic activity was determined using the Beer-Lambert law with a molar extinction coefficient of 1310 L mol-^1^ cm-^1^.

### 2.7 CETP activity

CETP activity was determined using a commercial assay kit (MAK106-1 KT, Merck, Darmstadt, Germany) following the manufacturer's protocol. Diluted serum samples were incubated with donor and acceptor molecules in a buffer at 37 °C for 3 h. The donor molecule contains a self-quenched fluorescent lipid, which exhibits increased fluorescence upon CETP-mediated transfer to the acceptor molecule. Fluorescence intensity was measured at an excitation wavelength of 465 nm and an emission wavelength of 535 nm.

### 2.8 Statistical analysis

Statistical analyses were conducted using SPSS (Version 29.0.0.0) (SPSS, Inc., Chicago, IL, USA) and GraphPad Prism 8.0. A p-value of less than 0.05 was considered statistically significant. Participant characteristics are represented as the median and interquartile range (Q1, Q3) or count and proportion. Mann-Whitney *U* Test or Fischer Exact Test were used to examine differences in clinical and laboratory characteristics, HDL composition, metabolism, and function between AHF patients who survived and those who died within 3 months after index AHF hospitalization. The prognostic value of HDL parameters for 3-month mortality was examined using univariable and multivariable Cox regression analyses. Kaplan-Meier survival analyses were performed to compare the tertiles of the HDL parameters using the log-rank test. The Spearman correlation coefficient was used to assess correlations between the measured indicators of HDL function and metabolism and HDL subclasses.

## 3 Results

### 3.1 Clinical characteristics of the study cohort

A total of 315 patients with severe clinical signs and symptoms of AHF requiring hospitalization were enrolled in the study over 36 months. The median age of the cohort was 76 years and 43 % of patients were female. More than 90 % of the enrolled patients had a prior diagnosis of cardiomyopathy and the index presentation was an acute worsening of chronic heart failure. At the time of presentation to the emergency department, all enrolled patients were in New York Heart Association (NYHA) functional class III (5.4 %) or IV (94.6 %). The demographic characteristics, comorbidities, vital signs, other physical measurements, and laboratory test results obtained at the time of presentation to the emergency department, as well as the classification of participants into different AHF groups, are presented in detail in Table 1 and Supplementary Table 1. Chronic medications of the AHF cohort are listed in Supplementary Table 2. Within three months after the index AHF hospitalization, 74 patients (23.5 %) died. Table 1 and Supplementary Table 1 also illustrate the differences between those who survived and those who succumbed within three months after the index AHF hospitalization. Patients who died within three months after the index AHF hospitalization were significantly older than those who survived the same period, and more likely to have concomitant chronic kidney disease and chronic pulmonary disease. The deceased patients had significantly higher body mass index (BMI), lower systemic blood pressure, higher pulmonary artery pressure, and greater left ventricular dilation. However, there was no significant difference in left-ventricular ejection fraction at the time of presentation to the emergency department due to AHF. In addition, patients who died within 3 months of the index hospitalization for AHF had significantly more elevated serum levels of NT-proBNP, more markedly impaired renal function, more reduced hepatic biosynthetic capacity (decreased cholesterol and albumin levels), and more elevated levels of inflammatory markers, including C-reactive protein and interleukin-6 (acute infectious disease was an exclusion criterion for inclusion). These measurements were obtained at the time of emergency department presentation.

**Table 1:** Baseline characteristics of the study cohort.

	Alive	Deceased	All	p-Value
	(n = 241)	(n = 74)	(n = 315)	
**Demographics**				
Age (years)	74.0 (66.0, 81.0)	79.0 (69.5, 85.8)	76.0 (67.0, 82.0)	**0.004**
Sex, Female	106 (44.0 %)	30 (40.5 %)	136 (43.2 %)	0.688
**Comorbidities**				
Hypertension	227 (94.2 %)	67 (90.5 %)	294 (93.3 %)	0.289
T1DM	2 (0.8 %)	2 (2.7 %)	4 (1.3 %)	0.236
T2DM	100 (41.5 %)	32 (43.2 %)	132 (41.9 %)	0.789
CAD	123 (51.0 %)	33 (44.6 %)	156 (49.5 %)	0.354
CMP	217 (90.0 %)	71 (95.9 %)	288 (91.4 %)	0.154
AF	122 (50.6 %)	48 (64.9 %)	170 (54.0 %)	**0.034**
CKD	94 (39.0 %)	49 (66.2 %)	143 (45.4 %)	**< 0.001**
COPD	57 (23.7 %)	27 (36.5 %)	84 (26.7 %)	**0.035**
MetS	164 (68.0 %)	53 (71.6 %)	217 (68.9 %)	0.667
**Physical examination at the time of presentation to the emergency department**				
BMI (kg/m2)	27.5 (24.8, 31.2)	29.4 (26.3, 33.0)	28.0 (25.0, 31.6)	**0.018**
MAP (mmHg)	103.3 (90.0, 120.0)	90.0 (82.1, 106.7)	100.0 (88.3, 118.3)	**< 0.001**
Heart rate (beats/min)	100.0 (80.0, 115.0)	95.5 (76.0, 118.8)	100.0 (80.0, 116.0)	0.185
Respiratory rate (breaths/min)	28.0 (24.0, 32.0)	28.0 (25.0, 34.0)	28.0 (24.0, 33.0)	0.336
**Laboratory parameters at the time of presentation to the emergency department**				
Total cholesterol (mmol/L)	3.7 (2.9, 4.7)	3.3 (2.8, 4.0)	3.5 (2.9, 4.5)	**0.008**
HDL-C (mmol/L)	1.1 (0.9, 1.4)	1.0 (0.8, 1.2)	1.1 (0.9, 1.3)	**0.027**
LDL-C (mmol/L)	1.9 (1.4, 2.8)	1.8 (1.4, 2.3)	1.9 (1.4, 2.7)	0.052
Albumin (g/L)	38.0 (35.2, 41.8)	36.6 (34.0, 39.2)	37.8 (34.8, 41.3)	**0.011**
Creatinine (μmol/L)	111.0 (87.0, 148.0)	133.5 (107.8, 168.0)	117.0 (90.5, 152.5)	**0.001**
eGFR (mL/min/1.73 m2)	51.0 (33.4, 68.8)	38.4 (29.3, 50.2)	46.6 (32.3, 65.0)	**< 0.001**
CRP (mg/L)	10.3 (4.7, 25.5)	31.2 (10.0, 55.4)	12.2 (5.5, 33.1)	**< 0.001**
IL-6 (pg/mL)	22.9 (11.6, 45.0)	58.3 (20.1, 104.8)	25.1 (12.9, 60.1)	**< 0.001**
NT-proBNP (pg/mL)	5796.0 (3315.0, 12323.0)	10568.0 (5855.0, 20537.8)	6692.0 (3531.0, 14395.5)	**< 0.001**
**AHF type**				0.154
New onset AHF	24 (10.0 %)	3 (4.1 %)	27 (8.6 %)	
AHF following CHF	217 (90.0 %)	71 (95.9 %)	288 (91.4 %)	
**NYHA class at the time of presentation to the emergency department**				0.378
3	15 (6.2 %)	2 (2.7 %)	17 (5.4 %)	
4	226 (93.8 %)	72 (97.3 %)	298 (94.6 %)	
**Echocardiography**				
LVEDd/BSA (mm/m2)	29.1 (18.0, 44.8)	27.4 (18.3–38.8)	28.5 (18.0, 44.8)	**0.043**
IVS (mm)	13.0 (2.0, 19.0)	13.0 (8.0, 22.0)	13.0 (2.0, 22.0)	0.189
PW (mm)	13.0 (8.0, 19.0)	13.0 (8.0, 16.0)	13.0 (8.0, 19.0)	0.313
LVEF (%)	40.0 (15.0, 66.0)	40.0 (15.0, 65.0)	40.0 (15.0, 66.0)	0.972
SPAP (mmHg)	50.0 (30.0, 90.0)	52.5 (30.0, 102.0)	50.0 (30.0–102.0)	**0.010**
**AHF class**				0.648
HFrEF, EF < 40 %	110 (46.6 %)	33 (49.3 %)	143 (47.2 %)	
HFmrEF, EF 41–49 %	66 (28.0 %)	15 (22.4 %)	81 (26.7 %)	
HFpEF, EF ≥ 50 %	60 (25.4 %)	19 (28.4 %)	79 (26.1 %)	

Participant characteristics are reported as median and interquartile range (Q1, Q3), as well as counts and frequencies. Differences between AHF patients who were alive and those who died within 3 months after index AHF hospitalization were tested using the Mann–Whitney *U* test or Fisher's exact test. P values < 0.05 are considered significant and are depicted in bold. AHF, acute heart failure; BMI, body mass index; CAD, coronary artery disease; CHF, chronic heart failure; CMP, cardiomyopathy; COPD, chronic obstructive lung disease; CRP, C-reactive protein; eGFR, estimated glomerular filtration rate; IL-6, interleukin-6; IVS, interventricular septum thickness; MAP, mean arterial pressure; HFrEF, heart failure with reduced ejection fraction; HFmrEF, heart failure with mildly reduced ejection fraction; HFpEF, heart failure with preserved ejection fraction; HDL-C, high-density lipoprotein cholesterol; LDL-C, low-density lipoprotein cholesterol; LVEDd, left ventricular end-diastolic diameter; LVEF, left ventricular ejection fraction; MetS, metabolic syndrome; NT-proBNP, N-terminal B-type natriuretic peptide; PW, left ventricular posterior wall thickness; SPAP, systolic pulmonary artery pressure; T1DM, type 1 diabetes mellitus; T2DM, type 2 diabetes mellitus.

### 3.2 Comparative analysis of baseline functionality, metabolism of HDL, and serum levels of HDL subclasses in AHF survivors versus non-survivors after 3-month follow-up

**Fig. 1:**
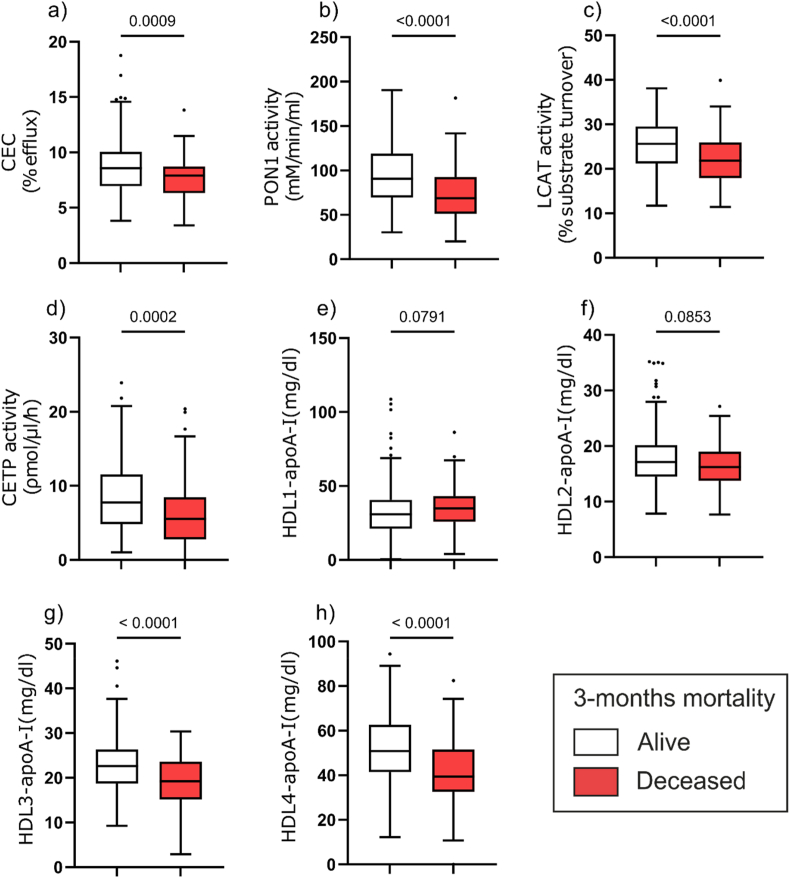
Differences in indicators of HDL serum levels, function, and metabolism between AHF patients who died compared to those who were alive within 3 months after index AHF hospitalization. CEC of HDL (a), HDL-associated PON1-activity (b), serum LCAT-activity (c), serum CETP-activity (d), HDL1-apoA-I (e), HDL2-apoA-I (f), HDL3-apoA-I (g), and HDL4-apoA-I (h), were assessed. Data are presented as Tukey box plots with median and interquartile range, as well as minimum, maximum, and outliers. Differences between groups were analyzed using the Mann-Whitney *U* test. P values < 0.05 were considered significant. ApoA-I, apolipoprotein A-I; CEC, cholesterol efflux capacity; CETP, cholesteryl ester transfer protein; HDL, high-density lipoprotein; LCAT, lecithin-cholesterol acyltransferase; PON1, paraoxonase 1.

In a comparative analysis, we observed that patients who died within three months after index AHF hospitalization had significantly altered functional, structural, and metabolic characteristics of HDL compared to survivors. Specifically, markers such as CEC, PON1 activity, and LCAT and CETP enzymatic activities were significantly reduced (Fig. 1). In addition, we observed a marked reduction in levels of the small HDL subclasses (HDL3-apoA-I and HDL4-apoA-I) in non-surviving patients, whereas levels of larger HDL subclasses (HDL1-apoA-I and HDL2-apoA-I) were not altered.

### 3.3 Associations of HDL subclasses, function, and metabolism with 3-month mortality after index AHF hospitalization

**Fig. 2:**
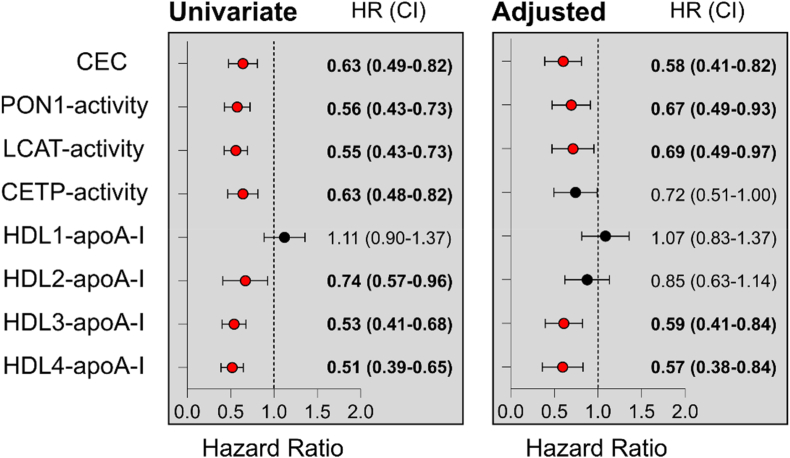
Cox regression analysis was used to assess the association between standardized HDL-related parameters and HDL subclasses and the risk of 3-month mortality following index AHF hospitalization. Hazard ratios (HRs) per 1 standard deviation increase and 95 % confidence intervals (CIs) were calculated. Covariates included age, sex, body mass index (BMI), smoking status, diabetes mellitus, total cholesterol, creatinine, interleukin-6 (IL-6), albumin, systolic blood pressure, diastolic blood pressure, and N-terminal pro-B-type natriuretic peptide (NT-proBNP). Significant associations (p < 0.05) are highlighted in bold. Abbreviations: ApoA-I, apolipoprotein A-I; CEC, cholesterol efflux capacity; CETP, cholesteryl ester transfer protein; HDL, high-density lipoprotein; LCAT, lecithin-cholesterol acyltransferase; PON1, paraoxonase 1.

Univariable Cox regression analyses showed that lower CEC, activities of PON1, LCAT, and CETP, as well as lower serum levels of small HDL subclasses were all significantly associated with an increased risk of 3-month mortality after an index AHF hospitalization (Fig. 2). These associations (with exception of HDL2-apoA-I and CETP activity) remained significant after adjustment for age, sex, BMI, smoking status, presence of diabetes, total cholesterol, creatinine level, interleukin-6 (IL-6), albumin, systolic and diastolic blood pressure, and NT-proBNP (Fig. 2).

**Fig. 3:**
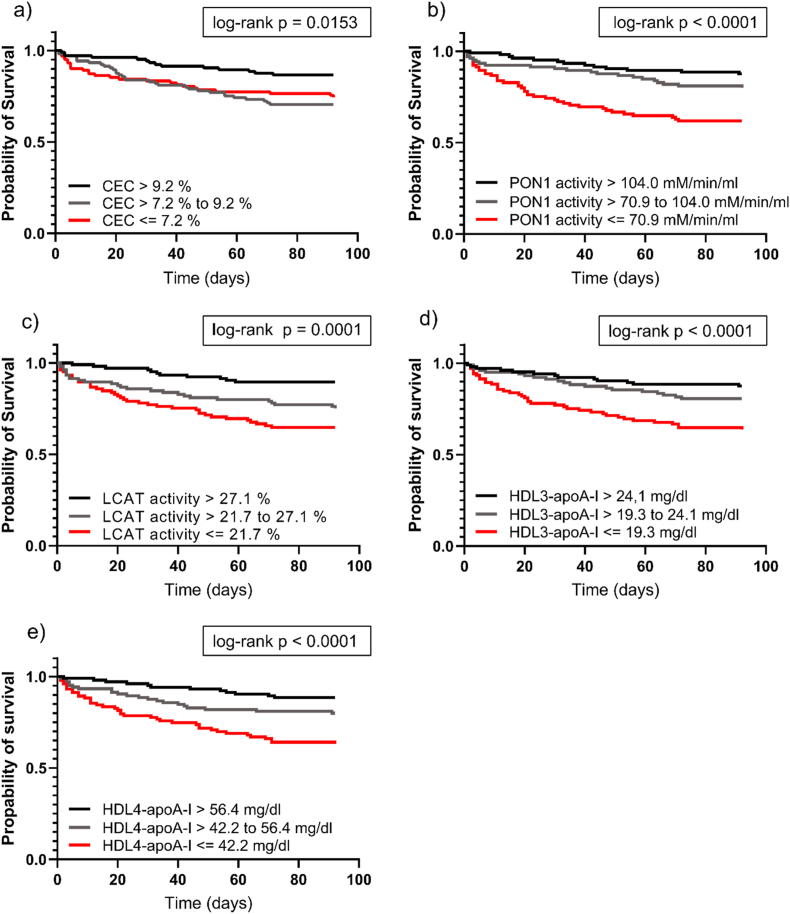
Kaplan-Meier 3-month survival estimates with subsequent log-rank test of HDL-related parameters. For each variable assessed, AHF patients (n = 315) were categorized into tertiles for CEC, (a), PON1-activity (b), LCAT-activity (c), HDL3-apoA-I (d), HDL4-apoA-I (e). Patients were divided into tertiles having high, middle, or low activities of enzymes or serum concentrations of apolipoproteins. Number of patients per tertile is 105. ApoA-I, apolipoprotein A-I; CEC, cholesterol efflux capacity; HDL, high-density lipoprotein; LCAT, lecithin-cholesterol acyltransferase; PON1, paraoxonase 1.

For Kaplan-Meier survival analysis (Fig. 3), patients were categorized into tertiles based on HDL parameters. Patients with the highest levels of CEC, PON1, and LCAT activities, as well as small HDL subclasses (HDL3-apoA-I and HDL4-apoA-I), demonstrated significantly better 3-month survival following index AHF hospitalization compared to those in the lowest tertile.

We performed receiver operating characteristics curve analyses to assess the accuracy of CEC, PON1 activity, LCAT activity, HDL3-apoA-I, and HDL4-apoA-I to predict mortality within 3 months after index AHF hospitalization (Supplemental Fig. 2). The best predictive value with an area under the curve (AUC) of 0.69 was observed for HDL3-apoA-I, followed closely by LCAT activity (AUC of 0.68), HDL4-apoA-I (AUC of 0.68) and PON1 activity (AUC of 0.67) and CEC (AUC of 0.63). All HDL parameters showed a comparable predictive value to NT-proBNP (AUC of 0.66).

### 3.4 Correlation between HDL function/metabolism and composition of HDL subclasses

**Fig. 4:**
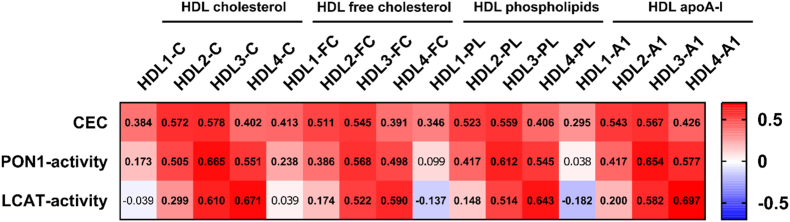
Heatmap for correlation analyses between indicators of HDL function and metabolism and serum levels of HDL subclasses. Values are presented as Spearman correlation coefficient. P-values <0.05 are considered significant after a Bonferroni correction for multiple testing and significant correlations are depicted in bold. A1, apolipoprotein A-1; C, cholesterol; CEC, cholesterol efflux capacity; FC, free cholesterol; HDL, high-density lipoprotein; LCAT, lecithin cholesterol acyltransferase; PL, phospholipid; PON1, paraoxonase 1.

Correlations between serum levels of HDL particles/subclasses and the indicators of HDL function and metabolism were examined by Spearman correlation analyses (Fig. 4). We found that CEC was significantly positively correlated with all HDL subclasses, however, most profoundly with parameters of medium-size HDL subclasses 2 and 3. In contrast, PON1-activity was most profoundly positively correlated with parameters of HDL subclass 3 followed by subclasses 4 and 2, and only weakly with large-buoyant HDL subclass 1. Similarly, as found for PON1-activity, the LCAT-activity was most profoundly positively correlated with small HDL subclasses 3 and 4.

## 4 Discussion

Previous research suggested that the antioxidative and anti-inflammatory properties of HDL may play a crucial role in the pathogenesis of HF by protecting the structure and function of the heart and thus reducing the risk of death [28]. Previous findings demonstrated that smaller HDL subclasses [2,29] and impaired HDL cholesterol efflux capacity [30] are associated with increased mortality risk following index AHF hospitalization. However, no studies have directly compared these metrics. Our study is the first comprehensive assessment of multiple measures of HDL function, structure, and metabolism in a cohort of patients with severe clinical signs and symptoms of AHF who presented to the emergency department and required hospitalization. Multivariable Cox regression analysis demonstrated that CEC, LCAT, and PON1 activity, as well as small HDL subclasses, remained significantly inversely associated with 3-month mortality after adjusting for multiple clinical and laboratory risk factors.

Interestingly, using ROC analysis we identified three promising predictors of mortality after hospitalization due to severe clinical signs and symptoms of AHF: PON1 activity, LCAT activity, and levels of small-density HDL subclasses. These markers performed similarly to the current gold standard for HF diagnosis and prognosis, NT-proBNP. Notably, small HDL subclasses can be conveniently measured by NMR, making them potentially suitable for routine clinical use [31].

While total HDL cholesterol levels have traditionally been used to assess cardiovascular risk, this approach may not capture the relevant information. NMR technology offers a more nuanced approach by allowing the analysis of HDL subclasses. This provides a deeper understanding of a patient's HDL particle function, as we observed a strong correlation of CEC, PON-1, and LCAT activities with small HDL subclasses. Biomarkers related to HDL function could provide complementary information and enhance risk stratification for mortality of the patients hospitalized due to severe signs and symptoms of AHF [32].

Our study has limitations. Due to the observational design, this study cannot establish causality between the observed associations. Reproducing our results across disparate cohorts, ethnicities, and earlier disease stages would enhance the overall external validity of these findings.

The present study offers several key strengths. First, it provides comprehensive insights into HDL's functional, structural, and metabolic properties and their relationship to outcomes in AHF patients. Unlike prior studies focusing on HDL structure, we comprehensively assessed HDL function, structure, and metabolism in hospitalized AHF patients, offering novel insights into HDL's role in this population. Notably, we observed a strong positive correlation between the functionality of HDL and its subclasses. Smaller HDL particles, particularly HDL3 and HDL4, exhibited a strong association with CEC, PON1 activity, and LCAT activity. These findings are consistent with previous research suggesting that the protective effect of higher HDL particle concentration on heart failure mortality is likely driven primarily by these smaller HDL subclasses [1,33]. By determining a patient's HDL subclasses, clinicians may be able to tailor treatment plans to target these specific HDL functions. This personalized approach could lead to more effective treatment of HF, especially in patients with cardiomyopathies in the early stages. HDL-mediated cholesterol efflux capacity could be a crucial protective mechanism in heart failure due to its potential to mitigate oxidative stress, inflammation, and apoptosis, all of which contribute to cardiac dysfunction. Large population studies have consistently linked reduced HDL cholesterol efflux capacity to increased cardiovascular risk. This reflects the anti-inflammatory and anti-atherogenic properties of cholesterol efflux pathways, which inhibit hematopoietic stem cell proliferation, macrophage inflammation, and foam cell formation [34]. By facilitating the removal of excess cholesterol from cardiac cells, HDL may help prevent lipid accumulation and cellular damage. In addition, HDL maintains endothelial function by promoting efflux of cholesterol and 7-oxysterols and preserving active eNOS dimer levels [22]. Supporting this notion, prior research has shown that administering a single bolus of reconstituted HDL after ischemia significantly enhanced myocardial glucose uptake and improved cardiac structural remodeling, leading to better functional recovery in mice [7]. It is important to highlight that sepsis is a significant contributor to mortality in individuals with heart failure, accounting for approximately 25 % of deaths in this population [35]. HDL exerts pleiotropic effects in host defense against pathogens, capable of sequestering and neutralizing potentially harmful substances like bacterial lipopolysaccharides and preventing viruses from entering or fusing with host cells [36].

Improving our understanding of HDL function and developing strategies to enhance its beneficial effects could lead to significant advancements in HF and AHF treatment.

## 5 Conclusions

Our findings suggest that specific HDL parameters could serve as valuable biomarkers for predicting severe AHF outcomes. This insight paves the way for the development of targeted therapies aimed at improving patient prognosis in the future.

## Funding

This research was funded by the Austrian Science Fund (10.13039/501100002428FWF) [Grant DOI 10.55776/DOC129] (doc. funds RESPImmun) and ApoA-I Mimetic Peptide Lipid Assemblies [Grant-DOI: 10.55776/I5703] and [Grant-DOI 10.55776/P28854, 10.55776/I3792, 10.55776/DOC130, and 10.55776/W1226], the 10.13039/501100004955Austrian Research Promotion Agency (10.13039/501100004955FFG) 864690 and 870454, the Integrative Metabolism Research Center Graz, the Austrian 10.13039/100031425Infrastructure Program 2016/2017, The Styrian Government (Zukunftsfonds, doc. funds program), the City of Graz, and BioTechMed Graz (Flagship project DYNIMO). For open access purposes, the author has applied a CC BY public copyright license to any author-accepted manuscript version arising from this submission.

## CRediT authorship contribution statement

**Anja Pammer:** Data curation, Methodology, Visualization, Writing – original draft, Writing – review & editing. **Iva Klobučar:** Data curation, Validation, Writing – review & editing. **Julia T. Stadler:** Formal analysis, Validation, Writing – review & editing. **Sabine Meissl:** Methodology, Writing – review & editing. **Hansjörg Habisch:** Data curation, Methodology, Writing – review & editing. **Tobias Madl:** Data curation, Funding acquisition, Writing – review & editing. **Saša Frank:** Data curation, Investigation, Writing – review & editing. **Vesna Degoricija:** Formal analysis, Resources, Writing – review & editing. **Gunther Marsche:** Conceptualization, Funding acquisition, Project administration, Supervision, Writing – original draft, Writing – review & editing.

## Declaration of Competing interest

The authors declare no conflicts of interest.
