# Supplementary material for: Impaired HDL antioxidant and anti-inflammatory functions are linked to increased mortality in acute heart failure patients
Source: Redox Biol. 2024 Sep 5;76:103341. doi: 10.1016/j.redox.2024.103341 (PMC11406013; doi:10.1016/j.redox.2024.103341)
Supplement: Multimedia component 1 [file mmc1.docx]

**Supplemental Material**

**
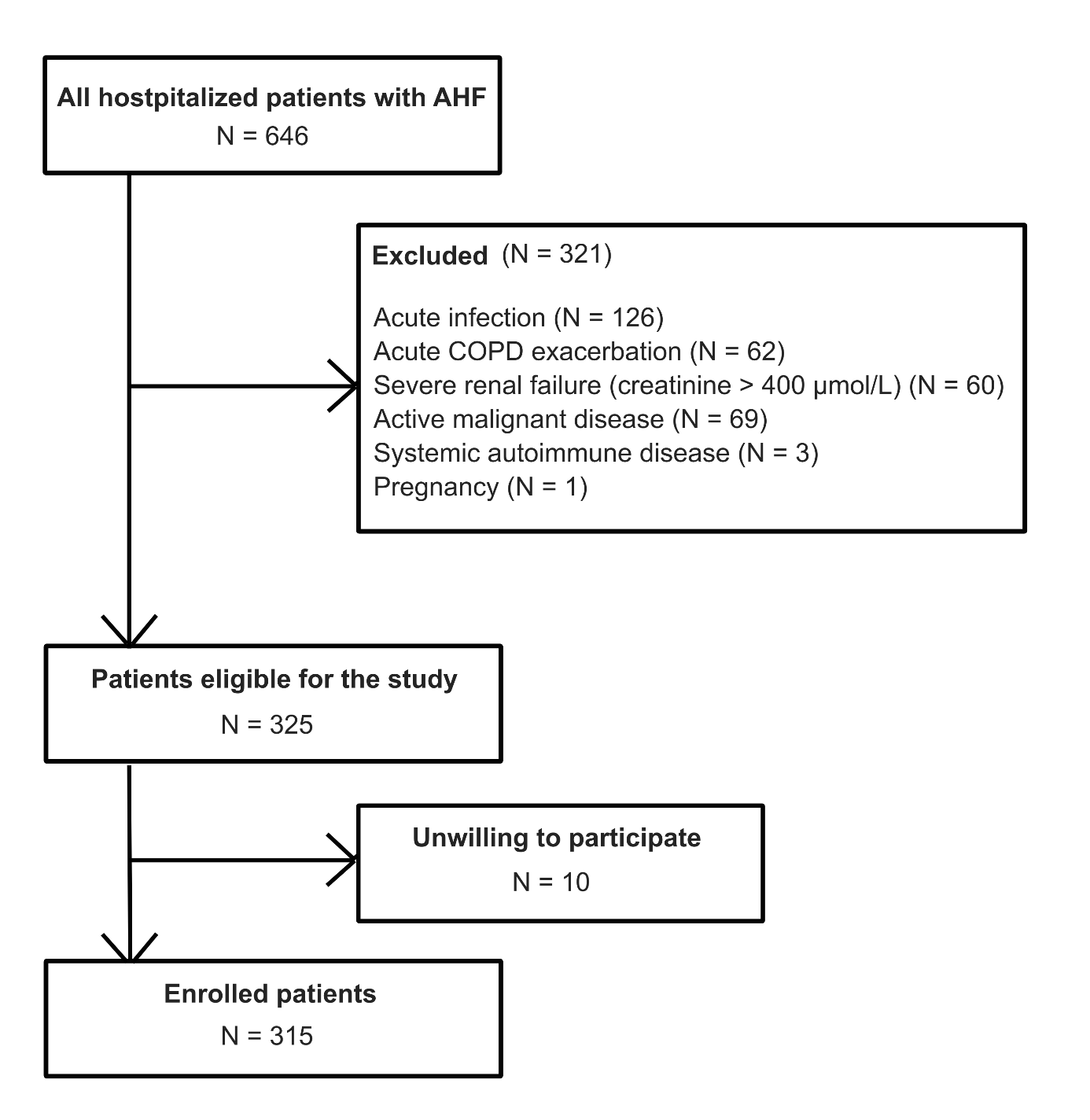
**

**Supplemental Figure 1** Study Flowchart. Exclusion criteria included age under 18 years, concomitant acute or chronic inflammatory disease, exacerbated chronic lung disease, severe renal insufficiency (serum creatinine ≥ 400 μmol/L), active malignancy, pregnancy, and refusal to participate. Of 646 AHF patients admitted to the Sisters of Charity University Hospital Centre in Zagreb, Croatia, between March 2018 and February 2021, 325 met the eligibility criteria for the study. Among them, 10 patients refused to participate.


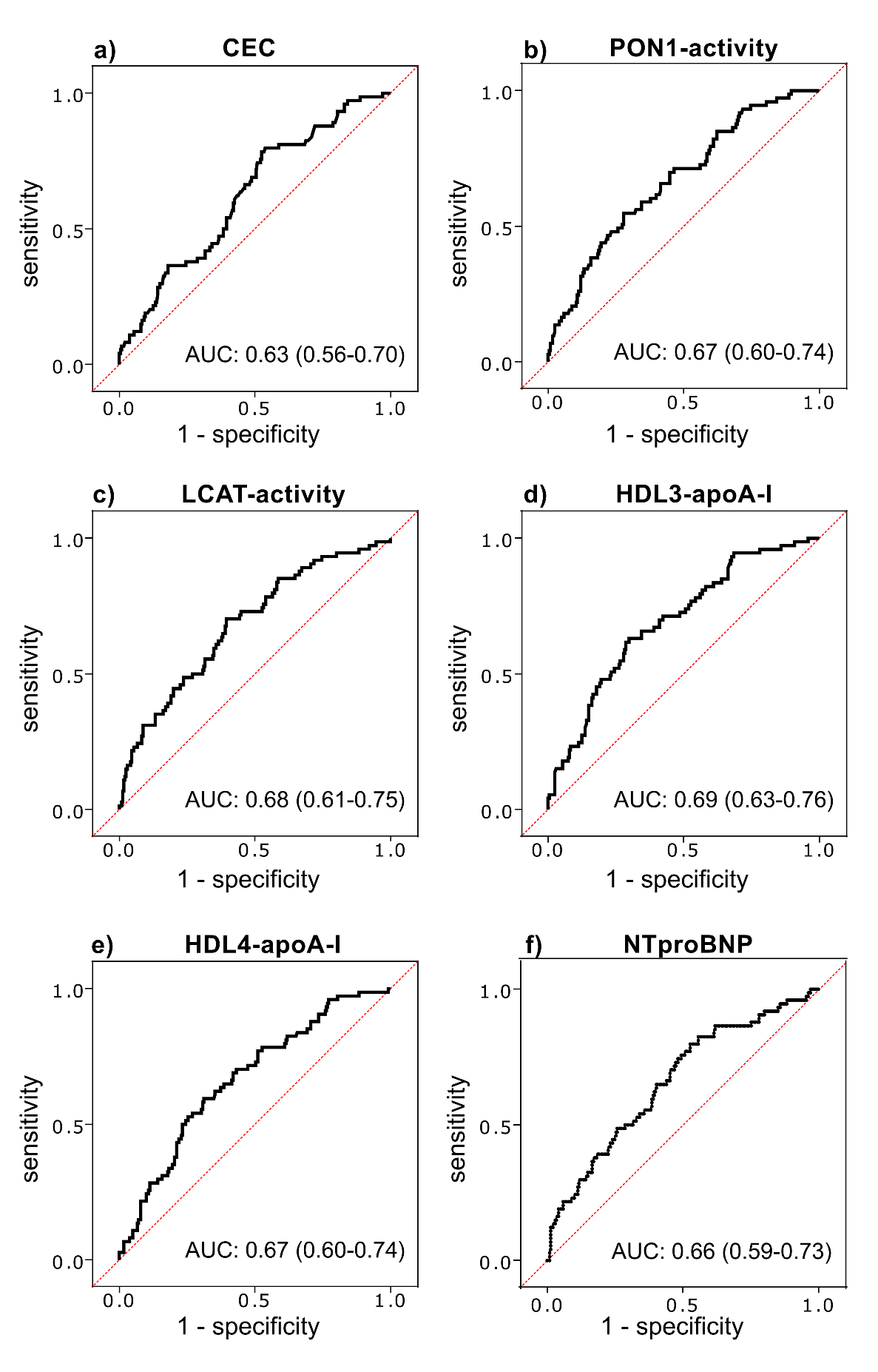


**Supplemental Figure 2** Receiver operating characteristics curves for prediction of death within 3 months after index AHF hospitalization. CEC (a), PON1 activity (b), LCAT activity (c), HDL3-apoA-I (d), HDL4-apoA-I (e), NTproBNP (f). ApoA-I, apolipoprotein A-I; CEC, cholesterol efflux capacity; HDL, high-density lipoprotein; LCAT, lecithin-cholesterol acyltransferase; NTproBNP, N-terminal pro-B-type natriuretic peptide; PON1, paraoxonase 1

**Supplemental Table 1.** Laboratory parameters at the time of presentation to the emergency department due to AHF.

|  | Alive | Deceased | All | p-Value |
| --- | --- | --- | --- | --- |
|  | (n = 241) | (n = 74) | (n = 315) |  |
| laboratory parameters | | | | |
| AST (U/L) | 27.0 (21.0, 42.0) | 28.0 (19.0, 56.0) | 28.0 (20.0, 44.5) | 0.982 |
| ALT (U/L) | 25.0 (16.0, 41.0) | 21.0 (13.0, 51.2) | 25.0 (15.0, 42.0) | 0.357 |
| CK (U/L) | 94.0 (59.0, 164.0) | 80.0 (51.8, 183.8) | 93.0 (58.0, 165.5) | 0.233 |
| LDH (U/L) | 253.0 (216.0, 315.0) | 289.5 (249.5, 407.5) | 265.0 (218.5, 332.0) | 0.003 |
| BUN (mmol/L) | 8.6 (6.6, 12.9) | 12.3 (9.0, 18.1) | 9.6 (6.9, 14.4) | < 0.001 |
| Sodium (mmol/L) | 140.0 (137.0, 142.0) | 138.0 (135.0, 140.0) | 140.0 (136.5, 142.0) | < 0.001 |
| Potassium (mmol/L) | 4.5 (4.1, 4.8) | 4.4 (4.1, 5.0) | 4.5 (4.1, 4.8) | 0.937 |
| Chloride (mmol/L) | 104.0 (100.0, 107.0) | 99.0 (96.0, 102.0) | 103.0 (99.0, 106.0) | < 0.001 |
| hsTnI (ng/L) | 39.0 (19.0, 130.0) | 70.0 (36.0, 178.0) | 46.0 (20.0, 143.2) | 0.002 |
| Triglycerides (mmol/L) | 1.0 (0.8, 1.3) | 1.0 (0.8, 1.2) | 1.0 (0.8, 1.3) | 0.390 |
| Erythrocytes (×1012/L) | 4.6 (4.3, 5.1) | 4.3 (3.8, 5.0) | 4.6 (4.2, 5.1) | 0.002 |
| Hemoglobin (g/L) | 137.0 (123.0, 149.0) | 123.0 (111.0, 138.8) | 134.0 (119.0, 148.0) | < 0.001 |
| INR | 1.2 (0.8, 5.3) | 1.4 (0.9, 6.1) | 1.2 (0.8, 6.1) | 0.008 |

Data are presented as median and interquartile ranges. Differences between AHF patients who were alive and those who died within 3 months after index AHF hospitalization were tested with the Mann–Whitney U test. p-values < 0.05 are considered significant and are depicted in bold. ALT, alanine aminotransferase; AST, aspartate aminotransferase; BUN, blood urea nitrogen; CK, creatine kinase; hsTnI, high-sensitivity troponin I; INR, international normalized ratio; LDH, lactate dehydrogenase;

**Supplemental Table 2** Chronic and acute medication of the AHF cohort

|  | Alive | Deceased | All | p-Value |
| --- | --- | --- | --- | --- |
|  | (n = 241) | (n = 74) | (n = 315) |  |
| chronic medication | | | | |
| Furosemide | 136 (56.4%) | 57 (77.0%) | 193 (61.3%) | 0.002 |
| Chlortalidone | 19 (7.9%) | 7 (9.5%) | 26 (8.3%) | 0.635 |
| Indapamide | 17 (7.1%) | 6 (8.1%) | 23 (7.3%) | 0.799 |
| MRA | 48 (19.9%) | 19 (25.7%) | 67 (21.3%) | 0.330 |
| ACEi | 127 (52.7%) | 42 (56.8%) | 169 (53.7%) | 0.595 |
| ATII | 29 (12.0%) | 4 (5.4%) | 33 (10.5%) | 0.129 |
| Entresto | 8 (3.3 %) | 0 (0 %) | 8 (2.5 %) | 0.205 |
| Amlodipine | 67 (27.8%) | 24 (32.4%) | 91 (28.9%) | 0.465 |
| Nitrate | 27 (11.2%) | 12 (16.2%) | 39 (12.4%) | 0.312 |
| Beta-blocker | 148 (61.4%) | 39 (52.7%) | 187 (59.4%) | 0.223 |
| Amiodarone | 22 (9.1%) | 3 (4.1%) | 25 (7.9%) | 0.219 |
| Digoxin | 18 (7.5%) | 10 (13.5%) | 28 (8.9%) | 0.158 |
| Statins | 96 (39.8%) | 23 (31.1%) | 119 (37.8%) | 0.217 |
| Fibrate | 1 (0.4 %) | 1 (1.4 %) | 2 (0.6 %) | 0.415 |
| SGLT2i | 7 (2.9 %) | 1 (1.4 %) | 8 (2.5 %) | 0.686 |
| GLPa | 0 (0 %) | 2 (2.7 %) | 2 (0.6 %) | 0.055 |
| Metformin | 40 (16.6%) | 12 (16.2%) | 52 (16.5%) | 1.000 |
| Sulphonylurea | 28 (11.6%) | 5 (6.8%) | 33 (10.5%) | 0.283 |
| DPP4i | 18 (7.5 %) | 6 (8.1 %) | 24 (7.6 %) | 0.806 |
| Thiazolidinediones | 4 (1.7 %) | 0 (0 %) | 4 (1.3 %) | 0.265 |
| Insulin | 36 (14.9%) | 15 (20.3%) | 51 (16.2%) | 0.282 |
| ASA | 60 (24.9%) | 23 (31.1%) | 83 (26.3%) | 0.295 |
| Warfarine | 61 (25.3%) | 23 (31.1%) | 84 (26.7%) | 0.368 |
| NOAC | 25 (10.4 %) | 3 (4.1 %) | 28 (8.9 %) | 0.107 |

Data are presented as n (%). Differences between AHF patients who died and those who survived the first three months after index AHF hospitalization were tested using Fisher’s exact test. ACEi, angiotensin-converting enzyme inhibitor; ASA, acetylsalicylic acid; ATII, angiotensin II receptor antagonist; DPP4i, dipeptidyl peptidase-4 inhibitor; GLPa, Glucagon-like peptide-1 receptor agonist; LMWH, low molecular weight heparin; MRA, mineralocorticoid receptor antagonist; NOAC, new oral anticoagulant; O2, oxygen; SGLT2i, Sodium–glucose cotransporter 2 inhibitor.
